# Psychopathological Implications of Behavioral Patterns in Obsessive–Compulsive Rituals: A Hierarchical Analysis

**DOI:** 10.3390/brainsci15060552

**Published:** 2025-05-23

**Authors:** Luca Gambolò, Anna Di Donna, Rebecca Ottoni, Stefano Parmigiani, Carlo Marchesi, Matteo Tonna

**Affiliations:** 1 Psychiatry Unit, Department of Medicine and Surgery, University of Parma, 43121 Parma, Italy; luca.gambolo@gmail.com (L.G.); anna.didonna01@gmail.com (A.D.D.); rebecca.ottoni@gmail.com (R.O.); carlo.marchesi@unipr.it (C.M.); 2 Local Health Agency of Piacenza, 29121 Piacenza, Italy; 3 Local Health Agency of Parma, 43125 Parma, Italy; 4 Unit of Behavioral Biology, Department of Chemistry, Life Sciences and Environmental Sustainability, University of Parma, 43121 Parma, Italy; stefano.parmigiani@unipr.it

**Keywords:** obsessive–compulsive disorder, rituals, biological evolution

## Abstract

**Background/Objectives**: Compulsive rituals in Obsessive–Compulsive Disorder (OCD) are characterized by a specific motor structure, built upon the fragmentation of action flow, obtained through act repetitions and the intrusion of non-functional acts. No study to date has adopted a hierarchical analysis to subtype OCD according to specific behavioral patterns, nor has a possible association between motor profiles and psychopathology been investigated. **Methods**: This study involved 31 OCD patients (11 female, 35%) and 31 healthy controls (11 female, 35%). The participants were asked to provide videotapes of their behaviors (OCD compulsions for patients and corresponding normal behaviors for healthy controls). BORIS software version 2.84.1 was adopted to analyze the recorded videos. Psychopathology was assessed through the Yale–Brown Obsessive–Compulsive Scale, the Childhood Trauma Questionnaire, the Frankfurt Complaint Questionnaire, and the Social and Occupational Functioning Assessment Scale. **Results**: Hierarchical analysis revealed three behavioral clusters based on motor profile: Cluster 1 included OCD compulsions structurally characterized by act repetitions (“iterative” rituals); Cluster 2 was represented by OCD compulsions mainly built upon non-functional acts (“idiosyncratic” rituals); and Cluster 3 comprised routinized and normative behaviors, without behavioral ritualization (no act repetitions and few non-functional acts). No significant differences were found in age, age at onset, and OCD severity between “iterative” and “idiosyncratic” rituals. However, patients with “iterative” rituals showed both more severe pre-psychotic symptoms and childhood trauma experiences than patients with “idiosyncratic” rituals. **Conclusions**: These findings may have significant clinical implications as they hint at a relationship between specific behavioral patterns of OCD compulsions and different underlying psychopathologies and/or vulnerabilities.

## 1. Introduction

Obsessive–Compulsive Disorder (OCD) is a disabling neuropsychiatric condition [1,2] that affects 1–3% of the general population [3,4]. It is characterized by the presence of obsessions (i.e., recurrent and persistent thoughts, urges, or images) and compulsions (i.e., repetitive behaviors or mental acts) aimed at reducing anxiety or preventing a feared event [5]. Obsessive–compulsive symptoms (OCSs) are time-consuming, cause significant distress, and interfere with everyday activities [5], eventually leading to impaired functioning and reduced quality of life [6].

OCD compulsions present a complex motor and cognitive structure, built on parceled action units, which may be “functional” (if they are necessary for the task at hand) or “non-functional” (if they are irrelevant or even unnecessary to the outcome) [7,8,9]. In particular, the following formal features allow to discriminate compulsive rituals from habitual behaviors and stereotypies: (1) OCD rituals are built on act repetitions, with a fragmentation of the motor flow [7,8,10,11]; (2) ritual compulsions involve the intrusion of non-functional acts in the motor performance, affecting its pragmatic aspects [7,9,10,11]; and (3) the attentional focus is shifted to these basic action units, reflecting an intentional and cognitive-demanding aspect in their execution (i.e., strict adherence to the ritual “script”) [12,13].

Different studies have highlighted the striking similarity between these specific motor parameters in OCD compulsions and those observed in human and non-human animal ritual behavior [11,13,14,15,16,17]. Ritual behavior may be defined as a specific way of organizing the flow of behavior, as first described by Rappaport [18] and subsequently developed in both anthropological and ethological fields [19]. Remarkably, OCD and ritual behavior share homologous features in terms of face validity (i.e., same formal structure) [20], construct validity (i.e., same neurobiological underpinnings, lying in the basal ganglia structures) [21], and predictive validity (as shown by robust animal models of OCD) [17]. Similar proximate mechanisms hint at underlying similar ultimate causations: from an ethological perspective, act repetition helps animals adjust their behavior in unpredictable ecological conditions [22,23,24], and would play a pivotal role in the development of motor behavior in vertebrates [11], whereas non-functional acts enhance behavioral flexibility to changing environments [8]. Across phylogeny, therefore, ritual appears as a highly evolutionarily conserved behavioral response to deal with conditions of potential danger [25], environmental unpredictability [13], or “high-entropy” state [26], that is, situations where individuals face chaotic or unpredictable inputs. Here, rituals may act as a compensatory mechanism, minimizing perceptual incoherence (i.e., restoring predictability and control), so as to counteract destabilizing environmental conditions [16].

Accordingly, in humans, OCD compulsions appear as specific behavioral outputs associated with different “high entropy” factors from childhood onwards, which may eventually affect normative developmental trajectories. Among these, the most studied are Childhood Trauma Exposures (CTEs) [27] and schizophrenia vulnerability, of which childhood OCSs may represent early biomarkers [28,29].

As to the former, up to 70% of people with CTEs develop OCSs later in life [30]. Moreover, a systematic review conducted by Destrée et al. (2021) showed that individuals with a history of CTEs tend to experience more severe OCSs [30]. In this connection, OCSs might initially represent an evolutionary developmental coping mechanism over early traumatic experiences [31,32], to regain a feeling of controllability and predictability over the environment [33], yet become increasingly dysfunctional over time [34].

As to schizophrenia vulnerability, the synchronic and/or diachronic co-occurrence of OCD and schizophrenia spectrum disorders is well documented in the literature [35,36,37]. The prevalence of OCD diagnosis in individuals with schizophrenia is 13.6% [38]. Moreover, in an 11-year follow-up study on 35,255 adolescents and adults with OCD [39], the progression rate from OCD to schizophrenia totaled 7.80%. OCSs/OCD can be detected throughout the course of the disease, including prodromal stages, first psychotic episodes, and chronic phases [40]. In this regard, mild/moderate OCSs appear to be associated with better levels of functioning in individuals with schizophrenia [41,42], in particular in everyday life and vocational functional domains [43]. These findings would corroborate the hypothesis that OCSs, particularly compulsive rituals, may confer a superimposed order and stability over the psychotic disorganizing process, sustaining work and daily life activities [43].

Remarkably, the very motor structure of OCD compulsions appears to move in concert with underlying psychopathology. The structural complexity of OCD compulsions (in terms of act repetitions, overall number of acts, and ritual duration), in fact, increases the severity of both trauma exposure and pre-psychotic symptoms in individuals with a primary diagnosis of OCD [7,44].

Therefore, the motor pattern of OCD compulsions seems to represent a dynamic response to different conditions of developmental unpredictability, either at a psycho-social (trauma events) or biological (psychotic vulnerability) level. As such, studying the specific motor structure of OCD compulsions might allow a more reliable sub-typing of compulsive phenotype in OCD based on objectifiable formal parameters, while offering a novel insight into their relationship with underlying psychopathology [9,10,12,45]. Moreover, a rigorous behavioral investigation of OCD compulsions encourages a cross-disciplinary dialogue within an evolutionary framework, between ethology and psychopathology, in the study of ritual behavior [46]. In this regard, the motor structure of OCD compulsions and how it diverges from normal behaviors has never been studied through a hierarchical analysis. The primary aim of this study is to investigate the hierarchical organization of compulsive behaviors in OCD and contrast their motor patterns with non-pathological counterparts. This approach allows us to identify distinct clusters of compulsive behaviors based on shared structural properties and pinpoint divergence points where pathological compulsions branch away from typical behaviors. We hypothesize that compulsions in OCD develop through divergent motor patterns, each underpinned by specific psychopathological conditions. By delineating these motor subtypes, we aim to link specific behavioral architectures to their psychopathological dynamics.

This study consists of two steps. The first step involves the investigation of motor behavior associated with various types of OCD compulsions in comparison to corresponding typical behavior. We will conduct a hierarchical analysis to identify potential unique motor patterns associated with OCD compulsions. The motor profile will be examined by considering two essential motor parameters: non-functionality and repetitiveness, which are supposed to constitute the building blocks of the ritualization process [15,16]. The second step aims to elucidate whether different motor subtypes of OCD compulsions may underlie specific psychopathological profiles (in terms of CTEs and pre-psychotic symptoms), to investigate possible relationships between compulsions’ motor structure and psychopathology in OCD.

Since there is evidence that behavioral traits are as reliable as morphological or molecular traits when inferring phylogenetic relationships [47,48], this exploration could also offer a framework for generating hypotheses concerning the evolutionary and developmental pathways of the motor structure of human compulsions through incremental behavioral changes from normal behavior.

## 2. Materials and Methods

### 2.1. Study Design

This study has a cross-sectional design and follows the STROBE (Strengthening the Reporting of Observational Studies in Epidemiology) statement [49]. We set a minimum sample size of 60 participants to ensure robust detection of the three hypothesized clusters in our hierarchical clustering analysis. This threshold was informed by heuristic guidelines for cluster analysis, which recommend 20 observations per cluster (60 total for 3 clusters) to avoid overfitting and ensure interpretability [50,51]. Additionally, a simulation-based power analysis (one-way ANOVA, α = 0.05, power = 0.8, Cohen’s f = 0.5) indicated a requirement of ≈52 observations to detect moderate between-cluster differences [52].

### 2.2. Participants

#### 2.2.1. OCD Patients

OCD individuals were outpatients recruited from the Psychiatric Unit of the University Hospital of Parma between July 2017 and July 2023. Patients were included in this study if (1) they were aged over 18 years; (2) received a diagnosis of Obsessive–Compulsive Disorder, according to DSM-5 criteria [5]; and (3) provided written informed consent to participate. Patients were excluded if they were affected by (1) a current affective or psychotic comorbidity; (2) a current mental disorder related to a general medical condition or a drug or alcohol abuse or dependence; or (3) a cognitive disorder (Mini-Mental State Examination score lower than 25), which could impair the compliance with testing procedures.

#### 2.2.2. Healthy Controls

Age and gender-matched healthy controls were recruited from January 2022 to July 2022 through advertisements among university personnel and students, without monetary compensation. In the announcement, potential participants were informed that they would be filmed while performing daily activities as part of a study. Participants were included in this study if they met the following criteria: (a) between 17 and 65 years of age; (b) provided written informed consent to participate. The exclusion criteria for healthy controls were (a) having a current mental disorder related to a general medical condition or to drug or alcohol abuse or dependence; and (b) having a cognitive impairment that could affect their ability to comply with the study requirements.

Before the enrolment, all participants were given a thorough and clear explanation of the study procedures, after excluding any conditions that could impair their understanding of the protocol and the questionnaire.

Both patients and healthy controls were asked to provide video recordings of their behaviors: respectively, OCD compulsions for patients and corresponding normal behaviors for healthy controls, as they spontaneously occurred in their daily lives. For example, if a patient described their ritual as “hand washing”, the respective control was requested to perform the same ordinary action. According to Zor and colleagues [10], we categorized acts as functional or non-functional based on whether they were necessary or irrelevant to task performance. Displaced acts, i.e., actions, that were functional per se but inserted out of context within the ritual script, were labeled as non-functional [44].

### 2.3. Treatment

All patients were treated with selective serotonin reuptake inhibitors [SSRIs] or clomipramine. Twenty-five patients (80.6%) were treated with SSRIs, and six patients (19.4%) were treated with clomipramine. Four non-responder patients (12.9%) received a second-generation antipsychotic augmentation. Responsiveness to OCD treatment was defined as a more than 35% reduction in the Yale–Brown Obsessive–Compulsive Scale (YBOCS) [53,54].

### 2.4. Sociodemographic and Clinical Assessment

Demographic information about participants was collected during enrollment through a specific schedule that included gender, age, educational level, and marital, occupational, and living statuses.

The Structured Clinical Interview for DSM-5 Clinician Version (SCID-5-CV) [55] confirmed the diagnosis of OCD. The SCID-5-CV is a semistructured interview guide for diagnosing major DSM-5 disorders, administered by a clinician familiar with DSM-5 criteria. It features diagnostic modules for mood disorders, psychotic disorders, substance use disorders, anxiety disorders, Obsessive–Compulsive Disorder and related disorders, eating disorders, somatic symptom disorders, sleep disorders (like insomnia and hypersomnia), externalizing disorders (such as intermittent explosive disorder, gambling disorder, and adult ADHD), and trauma- and stressor-related disorders.

The severity of OCD was measured with the Yale–Brown Obsessive–Compulsive Scale (YBOCS) [53]. YBOCS is a clinician-rated scale consisting of a symptom checklist to identify specific obsessions and compulsions and a severity scale that measures the intensity of these symptoms based on factors such as time consumption, distress, and interference with daily functioning. In our analysis, we specifically evaluated the total score and the obsession and compulsion subscales scores.

The total score of the Childhood Trauma Questionnaire (CTQ) [56] was adopted to assess the severity of childhood trauma experiences. The CTQ is a self-report instrument designed to assess experiences of childhood maltreatment and trauma. It consists of 28 items that evaluate five types of childhood trauma: emotional abuse, physical abuse, sexual abuse, emotional neglect, and physical neglect.

The Frankfurt Complaint Questionnaire (FCQ) [57] was used to assess pre-psychotic symptoms of schizophrenia. The FCQ is a self-report questionnaire specifically aimed at detecting basic symptoms (BSs) of schizophrenia, i.e., subtle disturbances experienced by the individual across various domains, including perception, thought processing, language, and attention preceding the onset of psychosis in schizophrenia spectrum disorders [58].

The Social and Occupational Functioning Assessment Scale (SOFAS) was used to assess the global functioning of the individuals. The SOFAS is a clinician-rated tool adopted to evaluate an individual’s overall social and occupational functioning level. It provides a single score ranging from 1 to 100, reflecting the degree of impairment in social relationships, work performance, and daily activities [59].

All assessments, including the SCID and various psychopathological rating scales, were conducted by a trained psychiatrist (RO). The clinical assessment took place over two sessions: the first session was dedicated to administering the SCID-5 CV, while the second session focused on administering and scoring the other clinical scales. After completing the questionnaires, participants were interviewed to assess the reliability of their responses. If there were any discrepancies or questionable answers, those questionnaires were deemed ineligible for this study. Additionally, participants who did not complete all the tests were excluded from the study sample.

### 2.5. Procedure

In the first session of this study, participants received a thorough explanation of the research and gave their consent to participate. The SCID-5 CV was then conducted to confirm or exclude a diagnosis of OCD. All participants completed a schedule to collect sociodemographic information. If there were any missing data, this schedule could be filled out during the second session. For the patient group, additional data were gathered from their clinicians.

Patients were subsequently asked to provide videotapes of their ritual behaviors as they occur throughout their daily lives. This could be submitted via a USB stick or sent to a specific email address. Patients were allowed to record the videotape by themselves or assisted by a relative. Patients were encouraged to showcase their most recent and frequently performed rituals. When asked to rate the similarity between the videotaped ritual and their off-camera compulsions, patients reported a moderate to high degree of similarity. This aligns with previous research [10,12], which indicated that once patients begin performing their rituals, they become absorbed in the act itself, paying no or little attention to the observer or the camera. Healthy control participants were instructed to demonstrate typical physiological behavior as it was performed in everyday life, such as how they wash their hands in their bathroom or how they close their front door. When asked to rate the similarity, healthy controls also reported a high degree of similarity between the videotaped behavior and their off-camera actions.

### 2.6. Behavioral Assessment

A ritual is defined as “the set of movements performed to accomplish a task as specified by the patient” [12]. This definition encompasses all the actions involved in the task. The beginning and the end of a ritual were determined by patients’ activities. Motor behavior was assessed while reviewing video recordings. We documented the actions that comprised each ritual. We used BORIS software to analyze the recorded videos [60]. This software automatically extracts key metrics that encompass the following motor features of behavior:

1. Total Number of Acts: The overall count of different actions performed during the ritual.
2. Number of Functional Acts: The count of actions carried out during the ritual that contribute to achieving its specific purpose.
3. Number of Non-Functional Acts: The count of actions that do not contribute to or are unrelated to the main goal of the ritual.
4. Total Duration: The overall length of the ritual from beginning to end.
5. Duration of Functional Acts: The total time spent performing actions that have a functional purpose within the ritual.
6. Duration of Non-Functional Acts: The time dedicated to actions that do not serve the purpose of the ritual.
7. Total Repetitions: The total number of acts performed throughout the ritual.
8. Repetitions of Functional Acts: The total count of repetitions of functional acts during the ritual.
9. Repetitions of Non-Functional Acts: The total count of repetitions of non-functional acts during the ritual.

### 2.7. Quantitative Analysis

We calculated the average and variability for continuous variables and the frequencies for categorical variables. To assess how often certain actions were repeated, we calculated the ratio of total repetition to the number of acts. We also computed the ratio between the number of repeated non-functional acts and the total repetition to measure non-functionality. Standardization of the index was implemented to achieve more accurate clustering. These ratios provided quantitative measures of repetitiveness and non-functionality in the observed behaviors.

### 2.8. Statistical Methods

All statistical analyses were performed using R version 4.4.2 [61].

### 2.9. First Step

Agglomerative cluster analysis explored the motor structure of compulsive and normal behaviors [62,63,64,65]. The percentage of normal behavior in each cluster was checked. ANOVA was used to evaluate differences in repetitiveness and non-functionality among patients’ clusters. Fisher’s Exact Test was adopted to identify a “normal behavior cluster” by analyzing the frequency of healthy individuals in each cluster.

### 2.10. Second Step

T-tests were conducted to assess differences in the severity of OCD (YBOCS total score), trauma exposure (CTQ total score), basic symptoms (FCQ score), as well as global functioning levels (SOFAS score), among clusters of ritualized behaviors.

## 3. Results

### 3.1. Participants

We enrolled 62 individuals: 31 OCD patients (11 female, 35%) and 31 healthy participants (11 female, 35%), matched for sex and age. Table 1 provides the clinical and sociodemographic characteristics of the study sample.

### 3.2. First Step: Motor Structure of Behavior and Hierarchical Analysis

Table 2 presents the characteristics of patients’ and controls’ motor behavior.

**Figure 1:**
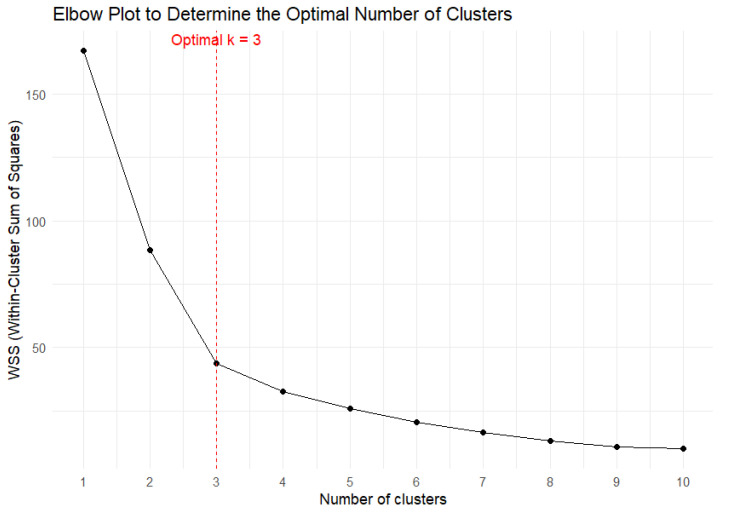
Elbow plot.

With respect to controls, patients exhibited an average longer duration of behavior, a higher number of total acts, non-functional acts, and a higher number of act repetitions. Figure 1 displays the elbow plot to determine the optimal number of clusters (k = 3).

**Figure 2:**
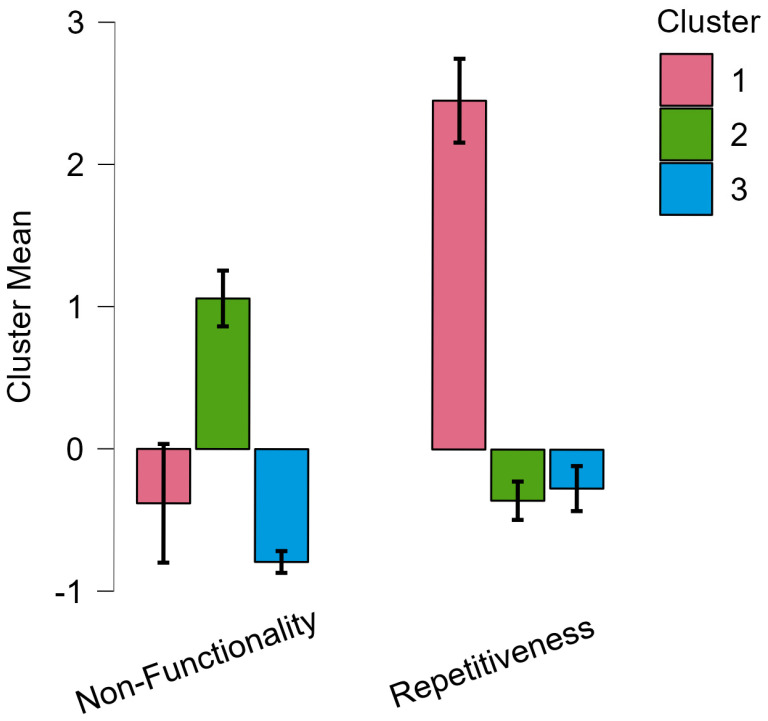
Non-functionality and repetitiveness among the clusters.

Figure 2 displays a graph showing non-functionality and repetitiveness among the clusters.

Table 3 reports the results of ANOVA and the number of OCD patients for every group.

**Figure 3:**
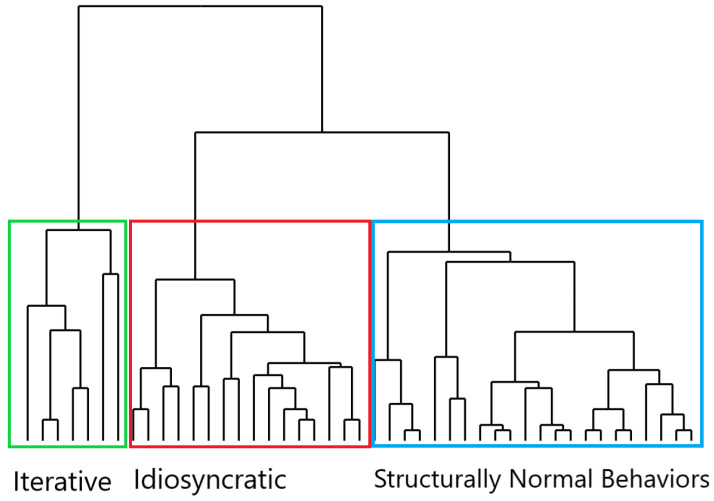
Cladogram of behaviors.

Hierarchical cluster analysis (Figure 3) identified three distinct behavioral clusters.

Cluster 1 included behaviors, mainly OCD compulsions, characterized by act repetitions, branching off at a higher level. Cluster 2, also primarily compulsions, featured behaviors mainly characterized by non-functional acts, which were separated at a lower level. Cluster 3 consisted of behaviors that showed no act repetitions and few non-functional acts. These behaviors belonged to both the OCD and control groups, even though only in the OCD group were they perceived as “compulsive-like” or mandatory. In the patients’ group, the lack of act repetition and non-functional acts differentiated them from rituals, making these behaviors more similar to routinized behavior (i.e., actions that are structurally more similar to normative behavior and are executed, according to rigid spatiotemporal parameters, during daily life activity [9,13]).

### 3.3. Second Step: Association Between Motor Structure of Behavior and Psychopathology

After excluding non-ritualized behaviors (including “routines”), ritual behaviors were collapsed into two subgroups: the “iterative” group (high repetitiveness and low non-functionality) and the “idiosyncratic” group (low repetitiveness and high non-functionality) [61]. Twenty-seven participants (male = 16, 59.3%) were included in the final step. All of them were represented by OCD compulsions. Among the reported rituals, 14 were identified as “idiosyncratic” (low repetitiveness and high non-functionality) (51.9%) and 13 as “iterative” (high repetitiveness and low non-functionality) (48.1%). Table 4 summarizes the sociodemographic characteristics and psychopathological dimensions, grouped by ritual type.

The analysis showed no significant differences in age, age at onset, or OCD severity (YBOCS total score) between the groups. The iterative group showed significantly higher severity of basic symptoms (FCQ score) and childhood traumatic experiences (CTQ total score) than the idiosyncratic group.

## 4. Discussion

The first part of the present study aimed at investigating how OCD compulsions might diverge in the motor pattern (in terms of repetitiveness and non-functionality) from corresponding normal behavior through a hierarchical analysis. Three major findings were derived from this study.

First, OCD compulsions exhibited a higher repetition of acts (both functional and non-functional) and a higher percentage of non-functional acts, with an overall broader range of total acts. This finding, in line with previous studies [7,9,10,11,12,44], confirms that OCD compulsions are built upon two partly distinct behavioral strategies: the repetition of acts and the intrusion of non-functional acts in the motor performance, overall leading to a heightened diversity of acts, and a fragmentation of the motor flow.

Second, after conducting a hierarchical analysis, we identified three distinct clusters, confirming our previous findings [50] but with different methodologies [66].

- Behavior with High Repetitiveness and Low Non-Functionality (Cluster 1): In this cluster, the motor performance was characterized by repetitions of acts (primarily functional ones) with a limited intrusion of non-functional acts in the action flow. These behaviors, centered on repetitive actions, were mainly displayed in the OCD group, and thus, they primarily represented OCD compulsions.
- Behavior with High Non-Functionality and Low Repetitiveness (Cluster 2): In this cluster, the ritual behavioral motor pattern was mainly built upon the intrusion of non-functional acts with little recourse to repetitive acts. Also, Cluster 2 behaviors were performed by OCD patients and might be identified as compulsions.
- Behavior with Low Repetitiveness and Low Non-Functionality (Cluster 3): In this cluster, the motor structure was not “ritualized”, as the behavioral performance was not fragmented into parceled units through repetitive acts and/or non-functional acts [20]. The majority of the controls’ behaviors were grouped within this cluster, thus forming a composition that primarily consisted of normal behaviors, although not exclusively. Indeed, OCD patients also exhibited Cluster 3 behaviors; in this case, however, the behavior, rather than “ritualized”, was more akin to “routines”, as it was stiffened by highly rigid spatiotemporal parameters [13]. For instance, an OCD patient might engage in handwashing routinized behavior similar to the controls’ behaviors in its formal structure (no repetitive acts and few non-functional acts), yet highly rigid and inflexible in the execution, so as to respect specific spatiotemporal features.

Third, hierarchical cluster analysis might suggest the underlying structural rearrangement from which the three distinct behavioral clusters stem. Cluster 1, characterized by highly repetitive behavior, branched off higher. These behaviors appear structurally closer to the common trunk, which may be considered as the “normative” or non-ritualized pattern of behavior. In contrast, the separation of non-functional behavior in Cluster 2 occurred at a lower level, whereas Cluster 3 behaviors are in structural continuity with the common trunk. Since hierarchical analysis represents a valid tool for reconstructing phylogenetic trees [62,67], as it allows to elucidate the relationships between different species based on shared characteristics (synapomorphies) [68], including behaviors [69,70,71], these findings might provide intriguing implications on the incremental structural adaptations of the motor pattern in ritual behavior, and in particular in OCD compulsions, during phylogeny.

In this respect, high-repetitiveness compulsions (Cluster 1) hint at a more original mechanism of ritualization, based on the repetition of species-specific behavioral patterns (e.g., “fixed action patterns”) [72], as they stem from the common trunk. Ethological studies suggest that ritual behaviors in non-human animals are mainly built upon the repetition of acts [13,17]. For example, the pecking courtship behavior in gallinaceous birds stems from the repetition of the act of feeding, expressed in an inflated and magnified form [73,74]. Consistently, in animal models of OCD, the repetition of functional acts is the most prevalent behavioral pattern, possibly indicating a more basic mechanism [11]. This perspective would be supported by the suggestion that the development of motor behavior in vertebrates follows “four phases” of recapitulation [11]. Particularly, studies on rat pup motor development show that behavior unfolds through a four-phase process: repetition of a first act (“repetition”), incorporation of a second act (“terminal addition”), decline in repetition of the first act (“condensation”), and performance of the second act without being preceded by the first act (“elimination”) [11]. This mechanism would explain the performance of highly repetitive motor patterns in response to changing environments in ethological accounts [33,75], as it allows to realign behavioral output to environmental mismatch [15]. Moreover, along with the development of higher cognitive abilities, act repetition has an anxiolytic effect by diverting attention from anxiety-related and stressful thoughts [20,76]. On the contrary, in OCD, iterative rituals would reflect a pathological failure to progress beyond the earlier phases of this sequence (repetition and addition) [11]. Thus, Cluster 1 could represent a specific subtype of OCD compulsions, with a specific motor structure resembling that of non-human rituals [15].

Instead, the incorporation of non-functional acts (Cluster 2) into the compulsive repertoire might suggest a further complexification of ritual motor structure from an evolutionary and developmental perspective. Non-functional acts have been described in non-human animal rituals (i.e., in companion animals) but are fully represented in human individual and collective rituals, including OCD compulsions [77]. These non-functional acts serve to disrupt automatic performance, enabling individuals to focus on motor execution and promoting a sense of control over unpredictable environments [50]. Moreover, they represent pivot points for adaptability, allowing individuals to tailor their actions to changing environments or whenever a mismatch occurs between behavioral output and environmental context [8].

Cluster 3 resulted in structural continuity with the common trunk, as their behaviors lacked significant repetitions with limited inclusion of non-functional acts, which are key aspects of ritualization [15,16]. Cluster 3 behaviors were mainly represented by normative behaviors, yet they also included OCD routines. Accordingly, routinized behavior is an important clinical feature of OCD, along with “typical” compulsive rituals, and may develop from rigid adherence to “just right” or sameness in daily routines [78,79].

The second part of this study aimed at investigating possible psychopathological differences among ritual subgroups. We found that OCD patients with iterative rituals (high repetitiveness and low non-functionality) had both more severe basic symptoms (FCQ total score) and past trauma experiences (CTQ total score), compared to those who displayed idiosyncratic rituals (low repetitiveness and high non-functionality). This result would suggest that heterogeneous sources of unpredictability, either at a biological or psycho-social level, occurring in developmental years, such as pre-psychotic symptoms or trauma exposure, would elicit a more original pattern of ritualization, which is based on act repetitions, with the homeostatic function of regaining a feeling of controllability and order [15,16].

Instead, idiosyncratic rituals would be preferentially recruited in “pure” OCD patients, that is, in primary forms of OCD not related to underlying divergent neurodevelopmental trajectories and/or comorbid conditions, marking, as we hypothesize, a further step in behavioral adaptation. In this case, the recourse of ritual behavior would be underpinned by a primary disruption in multisensory integration processing, as repeatedly highlighted in “pure” OCD patients [80,81,82].

The present results should be viewed with the caveat of the following limitations. The small sample size may affect the generalizability of the results; clusters derived from smaller datasets risk overfitting or reflecting noise rather than true behavioral distinctions. While participant-provided video recordings offered ecologically valid insights into ritualized behaviors, this approach introduced variability in recording conditions (e.g., self-recorded vs. assisted sessions, differences in lighting, camera angles, or environmental contexts) that may have influenced data quality. Although patients and healthy controls reported moderate-to-high similarity between recorded and real-world behaviors, self-recording could unintentionally alter naturalistic behavior. Furthermore, the lack of standardized recording protocols (e.g., fixed camera placement, controlled environments) limits direct comparability across participants. While our findings of structural OCD heterogeneity were primarily discussed under an evolutionary and psychopathological perspective, alternative explanatory frameworks—such as cognitive (e.g., dysregulated threat appraisal) or learning-based mechanisms (e.g., reinforcement schedules, habit acquisition)—could equally account for these patterns. In this regard, it should be noted that various approaches are not mutually exclusive, and all can help shed light on such a complex phenomenon from different angles. Moreover, the cross-sectional design of this study cannot rule out the possibility that the motor structure may change over time or have a phase-dependent effect. Finally, a potential confounding effect of psychopharmacological therapy on the compulsive motor profile was not taken into account. To address these limitations, future research should prioritize (1) larger, multisite cohorts to validate cluster stability and reduce overfitting risks inherent to hierarchical clustering in small samples; (2) standardized recording protocols (e.g., wearable cameras, fixed-angle guidelines) to balance ecological validity with methodological rigor; (3) longitudinal designs to track how ritual motor patterns evolve with symptom progression or treatment; (4) integration of cognitive and learning mechanisms (e.g., threat appraisal biases, habit reinforcement) to disentangle their role in ritual differentiation alongside evolutionary and developmental models; and (5) controlled investigations of psychopharmacological effects on motor profiles, including medication-naïve cohorts. Building on our observation that iterative rituals associate with neurodevelopmental perturbations (e.g., trauma, psychosis risk), future research should also explore specific biomarkers (e.g., cortico-striatal connectivity) of ritual subtypes.

The strength of this study lies in its pioneering approach. To the best of our knowledge, no studies to date have applied a hierarchical cluster analysis to explore the specific motor structure of OCD compulsions and how they differentiate from normal behaviors. Additionally, this model might offer important clinical implications, providing a broader perspective on the evolutionary and developmental underpinnings of OCD compulsions.

## 5. Conclusions

Iterative and idiosyncratic compulsions represent alternative behavioral strategies to cope with different sources of unpredictability. The former are triggered by early neurodevelopmental perturbations, such as psychotic vulnerability and trauma, whilst the latter are inherent to primary OCD and recruited in response to the unbalanced sensory grounding of the disorder. The present findings could have important clinical implications as the very motor structure of OCD compulsions might hint at different underlying psychopathological vulnerabilities and/or comorbid neurodevelopmental pathways, masked by more prominent obsessive–compulsive symptomatology.

## Figures and Tables

**Table 1:** Characteristics of the sample.

	Patients N = 31	Controls N = 31	*p*
**Female N (%)**	11 (35)	11 (35)	1
**Age, years M (SD)**	44.9 (20.1)	38.5 (15.8)	0.173
**SOFAS M (SD)**	65.6 (16.4)	86.0 (4.0)	<0.001
**YBOCS M (SD)**	21.4 (7.6)	1.1 (1.7)	<0.001
**FCQ M (SD)**	25.0 (23.6)	4.1 (2.8)	<0.001
**CTQ M (SD)**	34.9 (13.7)	27.7 (1.8)	0.005

**Table 2:** Formal structure of behaviors.

	Patients (N = 31)	Controls (N = 31)	*p*
**Duration (s) M (SD)**	48.9 (55.9)	9.8 (10.0)	<0.001
**Total Number of Acts M (SD)**	10.5 (6.9)	5.3 (4.3)	<0.001
**Number of Functional Acts M (SD)**	7.8 (6.8)	5.2 (4.2)	0.08
**Number of Non-Functional Acts M (SD)**	2.8 (2.6)	0.1 (0.3)	<0.001
**Total Repetitions M (SD)**	32.6 (34.9)	9.3 (10.7)	<0.001
**Repetitions of Functional Acts M (SD)**	24.5 (31.5)	9.3 (10.6)	<0.015
**Repetitions of Non-Functional Acts M (SD)**	8.1 (9.7)	0.1 (0.3)	<0.001
**Repetitiveness M (SD)**	2.7 (1.6)	1.4 (0.6)	<0.001
**Non-Functionality M (SD)**	0.4 (0.3)	0.2 (0.2)	<0.001

**Table 3:** Number of patients and the motor structure of behavior among clusters.

	Cluster 1 (*n* = 7)	Cluster 2 (*n* = 25)	Cluster 3 (*n* = 30)	One-Way ANOVA with Bonferroni Post Hoc Analysis
**OCD N (%)**	7 (100)	18 (72)	6 (20)	*p*
**Repetitiveness M (SD)**	5.5 (0.7)	1.6 (0.6)	1.7 (0.7)	<0.001 a,b
**Non-Functionality M (SD)**	0.2 (1.07)	0.6 (0.2)	0.1 (0.1)	<0.001 c,d

a. Cluster 1 > Cluster 2, *p* < 0.001. b. Cluster 1 > Cluster 3, *p* < 0.001. c. Cluster 2 > Cluster 3, *p* < 0.001. d. Cluster 2 > Cluster 1, *p* < 0.001.

**Table 4:** Comparison between iterative (Cluster 1) and idiosyncratic (Cluster 2) rituals in sociodemographic and psychopathological variables.

	Iterative (*n* = 13)	Idiosyncratic (*n* = 14)	*p*
**Age (y) M (SD)**	50.0 (19.4)	40.9 (21.2)	0.277
**Onset Age(y) M (SD)**	24.0 (12.2)	29.6 (12.3)	0.266
**Female N (%)**	7 (53.8)	4 (28.6)	0.182
**YBOCS M (SD)**	21.4 (9.3)	18.1 (8.9)	0.363
**SOFAS M (SD)**	63.5 (15.9)	65.0 (16.4)	0.807
**FCQM (SD)**	35.4 (27.7)	17.0 (15.2)	**0.048**
**CTQ**	42.3 (13.1)	33.4 (6.1)	**0.039**

Results in bold are statistically significant.

## Data Availability

The data presented in this study are available on request from the corresponding author due to privacy reasons.
